# Profile of elderly patients receiving antiretroviral therapy at Newlands Clinic in 2020: A cross-sectional study

**DOI:** 10.4102/sajhivmed.v21i1.1164

**Published:** 2020-12-10

**Authors:** Cleophas Chimbetete, Tinashe Mudzviti, Tinei Shamu

**Affiliations:** 1 Newlands Clinic, Harare, Zimbabwe; 2 School of Pharmacy, College of Health Sciences, University of Zimbabwe, Harare, Zimbabwe; 3 Institute of Social and Preventive Medicine, University of Bern, Bern, Switzerland

**Keywords:** HIV infection, elderly patients, comorbidities, ART, Zimbabwe

## Abstract

**Background:**

People living with HIV (PLWH) face new challenges such as accelerated ageing and higher rates of comorbidities including cardiovascular, renal and metabolic diseases as they age.

**Objectives:**

To profile the demographic and clinical characteristics of elderly patients receiving HIV care at Newlands Clinic (NC), Harare, Zimbabwe, as of 01 October 2019.

**Methods:**

A cross-sectional analysis was conducted using clinic data. All patients who were 50 years and older on 01 October 2019 were enrolled. Descriptive statistics (medians, interquartile ranges [IQRs] and proportions) were used to describe patient demographic and clinical characteristics.

**Results:**

Out of 6543 patients undergoing care at NC, 1688 (25.8%) were older than 50 years. The median duration of antiretroviral therapy (ART) was 10.9 years (IQR: 7.1–13). Over 90% of all patients had an HIV viral load below 50 copies/mL. Women were more likely than men to be overweight and obese (32% and 25% vs. 18% and 7%, respectively). Hypertension (41.2%), arthritis (19.9%) and chronic kidney disease (11.6%) were common comorbidities differently distributed based on sex. The most common malignancy diagnosed in women was cervical intra-epithelial neoplasia (68% of cancer burden in women) and Kaposi sarcoma was the leading malignancy in men (41% of cancer burden in men). Nearly 20% of patients had at least two chronic non-communicable comorbidities and 5.6% had at least three.

**Conclusion:**

A high burden of comorbidities was observed amongst HIV-positive elderly patients receiving ART. Age-appropriate monitoring protocols must be developed to ensure optimum quality of care for elderly HIV-positive individuals.

## Introduction

Effective antiretroviral therapy (ART) has changed the course of human immunodeficiency virus (HIV) infection from being considered a fatal illness to a chronic manageable condition. This has enabled people to grow old living with HIV. The number of people who are more than 50 years old and living with HIV has increased globally because of the success of national ART programmes and the control of new infections amongst the elderly.^1^ The life expectancy of people living with HIV (PLWH) has increased substantially, approximating that of the general population in high-income countries.^2,3^ Similar gains in life expectancy have also been reported in some countries in sub-Saharan Africa.^4^ It seems likely that the median age of PLWH will increase with time as more and more people garner the survival benefits of effective ART. It is now critical to promote healthy ageing amongst PLWH, an achievement that some have described as the ‘fourth 90%’.^5^

As PLWH age, they face many of the same health challenges as faced by older individuals in the general population. Additionally, ageing PLWH face new challenges such as accelerated ageing and higher rates of comorbidities including cardiovascular, renal and metabolic diseases.^6^ Comorbidities occur at high rates at all ages amongst PLWH but become increasingly important as this population ages. People living with HIV also develop geriatric syndromes and frailty earlier as compared to HIV uninfected people.^7^ Certain ART drugs such as protease inhibitors may increase cardiovascular risk because of poor metabolic profile including hyperlipidaemia compared to other drugs such as the non-nucleoside reverse transcriptase inhibitors.^8^ There has been a documented increase in the rate of all cancers, particularly lung, prostate, colorectal, anal, cervix, lymphomas and breast as PLWH age.^9^ However, it is important to note that cancer rates increase with age in everyone.

Although there is a growing body of research on HIV and ageing, evidence from low- and middle-income countries that bear the highest burden of HIV infection is scarce.^10,11^ The expectations and management of elderly PLWH differ from those of younger patients and may change over time. In this study, we profiled the demographic and clinical characteristics of elderly patients receiving comprehensive HIV care at Newlands Clinic (NC), Harare, Zimbabwe, as of 01 October 2019. There is evidence that the distribution of comorbidities may differ by sex^12^; therefore, we analysed and reported our results by sex.

## Methods

### Study design

This study utilised a cross-sectional analysis of routinely collected electronic clinic data.

### Study setting

The study was conducted at Newlands Clinic, Harare, Zimbabwe. Newlands Clinic, operated by the Ruedi Luethy Foundation, is a model public–private partnership with the Ministry of Health and Child Care. Since 2004 the clinic has offered comprehensive HIV treatment and care to individuals living with HIV. Operations of the clinic have been previously described.^13^ The clinic’s electronic database captures patient-level data on laboratory test results, diagnoses, medications and demographic variables.

### Sampling

Patients ≥ 50 years were defined as elderly. All patients who were 50 years and older as on 01 October 2019 were included in the analysis. The study had no exclusion criteria for enrolment.

### Data analysis

Routinely collected electronic patient data were abstracted from the clinic’s electronic database using custom structured language queries and exported to Excel. Records were de-identified at abstraction and assigned numeric unique identifiers. Stata version 13 was used for analysis. The most recent CD4 cell count and HIV viral load (VL) were defined as the last test result documented in the patient’s clinic notes taken within the preceding year. Weight categories were defined using body mass index (BMI) as follows: underweight (BMI < 18 kilogram/square meter [kg/m^2^]), normal weight (BMI of 18 kg/m^2^ – 25 kg/m^2^), overweight (BMI > 25 kg/m^2^ – 30 kg/m^2^) and obese (BMI > 30 kg/m^2^). Baseline age was defined as age at the date of initiating ART. Descriptive statistics (medians, interquartile ranges [IQRs] and proportions) were used to describe patient demographic and clinical characteristics. Diagnosis of comorbidities was conducted based on the following existing national guidelines: diabetes mellitus (DM) is defined as either fasting blood sugar ≥ 7.0 millimoles per litre (mmol/L) or random blood sugar of ≥ 11 mmol/L, if it still remains high after 3 months, then treatment is instituted. Hypertension is defined as two blood pressure readings > 140/90 millimetre of mercury (mmHg) at least 6 h apart. Chronic kidney disease (CKD) is defined as a serum creatinine clearance of < 60 milligrams per deciliter (mg/dL) confirmed with a second result within 3–6 months. All cancers ever diagnosed were included in the analysis and the pre-cancer conditions of vulva and cervical intra-epithelial neoplasia (VIN and CIN) were included as well.

Weight and height measurements were performed by nurses in the consulting rooms using Seca 752 or Seca 952 scales and wall-mounted height measure tapes, respectively. Before being weighed, patients removed shoes, jackets and emptied their pockets. Samples for blood glucose measurement were collected into 4-mL BD Vacutainer Plus plastic tubes with 10 mg sodium fluoride and 8 mg potassium oxalate additives. Blood samples for creatinine measurement were collected into 4-mL BD Vacutainer Plus plastic serum tubes with clot activator and silicone-coated interior. Glucose and creatinine measurements were conducted at the on-site laboratory on a COBAS Integra 400 plus instrument using the hexokinase and Jaffe reaction methods, respectively.

### Ethical consideration

Permission to carry out the study was granted by the Newlands Clinic local research board. Ethical approval was granted by the Medical Research Council of Zimbabwe, approval number: A1336. Participants consented to publication as part of the informed consent process.

## Results

Of the 6543 patients in care at NC, 1688 (25.8%) were above the age of 50 years (Table 1). The median age at analysis was 56 years (IQR: 52–61), with 72.2% of elderly patients aged between 50 and 60 years, and no difference by sex. The median duration of ART was 10.9 years (IQR: 7.1–13). At enrolment into care, 51% were married, 32% widowed, 6% divorced and 11% single. There was a far lower proportion of married women than married men (31% vs. 82%). The most recent median CD4 cell count in this cohort was 464 cells/mm^3^ (IQR: 327–628), which was higher in females than males (526 vs. 377). Over 90% of all patients were virally suppressed, with VLs below 50 copies/mL. Only 1.8% of patients had a VL > 1000 copies/mL. No sex differences were observed in the VL distribution. Almost half of the elderly patients were either overweight or obese and 8% were underweight. Women were more likely than men to be overweight and obese (32% and 25% vs. 18% and 7%, respectively). Most patients (64.1%) received the fixed dose ART combination of tenofovir, lamivudine and dolutegravir.

**TABLE 1:** Demographic and clinical characteristics of elderly patients receiving antiretroviral therapy at Newlands Clinic in March 2020, by sex.

Characteristic	Females		Males		Total	
	*n* = 1008	%	*n* = 680	%	*N* = 1688	%
**Baseline age in years, median (IQR)**	46.5	41.2–51.4	46.8	42.7–52.1	46.6	42.1–51.8
**Current age (years), median (IQR)**	56	52–61	56	52–61	56	52–61
**Current age in years, *n* (%)**						
50–60	729	72	491	72	1220	72
> 60–70	246	25	158	23	404	24
> 70	33	3	31	5	64	4
**Marital status at enrolment**						
Married	308	31	556	82	864	51
Widowed	478	47	69	10	547	32
Divorced	74	7	27	4	101	6
Single	148	15	28	4	176	11
CD4 count in cells/µL, median (IQR)	526	390–689	377	390–689	464	327–628
**HIV viral load expressed as RNA copies/mL, *n* (%)**						
< 50	939	93	617	91	1 756	92
50–1000	58	6	39	6	97	6
> 1000	9	1	21	3	30	2
**Body mass index category, *n* (%)**						
Underweight	48	5	88	13	136	8
Normal	380	38	406	60	786	47
Overweight	319	32	133	18	452	27
Obese	251	25	48	7	299	18
ART duration, years, median (IQR)	11.3	8.1–14.1	10.4	8.1–14.1	10.9	7.1–13
**Current ART regimen, *n* (%)**						
NNRTI† +2NRTI	144	14	97	14	241	14
TLD‡	671	67	411	60	1 082	64
ATV/r§ or LPV/r¶ + 2NRTI	171	17	143	21	314	19
DTG†† + Darunavir + ≤ 2NRTI	17	2	28	4	45	3
Other regimens	5	0.5	1	0.2	6	0.3

ART, antiretroviral therapy; IQR, interquartile range; HIV, human immunodeficiency virus; RNA, ribonucleic acid; NNRTI, non-nucleoside reverse transcriptase inhibitors; NRTI, nucleoside reverse transcriptase inhibitor; TLD, tenofovir, lamivudine and dolutegravir; ATV/r, atazanavir/ritonovir; LPV/r, Lopinavir/ritonovir; DTG, dolutegravir.

† , Either nevirapine or efavirenz.

‡ , Tenofovir, lamivudine and dolutegravir.

§ , Atazanavir/ritonavir.

¶ , Lopinavir/ritonavir.

†† , Dolutegravir.

### Comorbidities and pill burden

**FIGURE 1:**
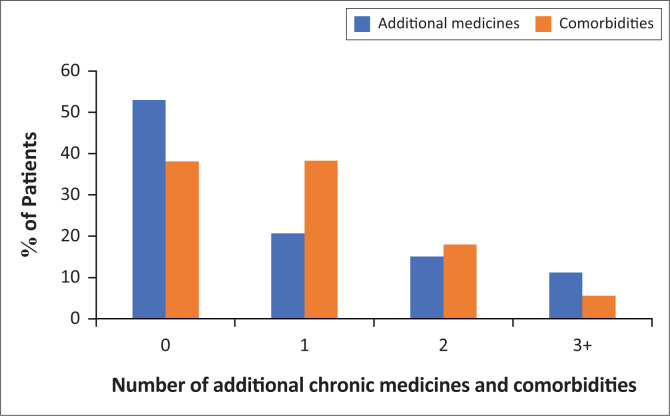
Distribution of comorbidities and additional chronic medicines amongst elderly patients receiving antiretroviral therapy at Newlands Clinic in March 2020.

Hypertension, arthritis and any malignancy were common comorbidities differently distributed by sex (Table 2). Hypertension was the most common comorbidity, with 704 (41.2%) patients having a documented diagnosis of hypertension. A higher proportion of women as compared to men had hypertension (48.5% vs. 31.6%, respectively). Arthritis was the second most common comorbidity and higher amongst women compared to men (24.5% vs. 13.1%), as was any malignancy (11.2% compared with 2.5%). Other comorbidities included DM (7.2%), CKD (11.6%) and congestive cardiac failure (2.2%), which were similarly distributed in men and women. Nearly 20% of patients had at least two chronic non-communicable comorbidities and 5.6% had three (Figure 1). Almost half of the cohort (47%) received an additional chronic medicine other than ART.

**TABLE 2:** Distribution of comorbidities amongst elderly patients receiving antiretroviral therapy at Newlands Clinic in March 2020, by sex.

Comorbidity	Females		Males		Total	
	*n* = 1008	60%	*n* = 680	40%	*n* = 1680	100%
Hypertension	489	48.5	215	31.6	704	41.2
Diabetes mellitus	68	6.8	53	7.8	117	7.2
Chronic kidney disease†	108	10.7	87	12.8	195	11.6
Any malignancy	113	11.2	17	2.5	130	7.7
Congestive cardiac failure	27	2.7	10	1.5	37	2.2
Arthritis	247	24.5	89	13.1	336	19.9
Tuberculosis	187	19	176	26	363	22

† , Chronic kidney disease = glomerular filtration rates of <60 mL/min/1.73 m^2^.

### Burden of malignancies ever diagnosed

Amongst women, the most common malignancy diagnosed was CIN3, accounting for 68% of the malignancy burden (Table 3). Other common malignancies in women included cancer of the cervix (12%) and VIN (9%). Amongst men, the most common malignancy was Kaposi Sarcoma (41%), followed by lymphomas and penile cancer (24%).

**TABLE 3:** Distribution of malignancies amongst elderly patients receiving antiretroviral therapy at Newlands Clinic in March 2020, by sex.

Malignancy type	Female		Male		Frequency	
	*n* = 113	%	*n* = 17	%	*n* = 130	%
CIN 3	77	68	-	-	77	59
Cancer of the cervix	14	12	-	-	14	11
Kaposi sarcoma	4	4	7	41	11	9
VIN 3	10	9	-	-	10	8
Lymphomas†	3	3	4	24	7	5
Penile cancer	-		4	24	4	3
Vulvar cancer	3	3	-	-	3	2
Multiple myeloma	1	1	1	6	2	2
Anal cancer	1	1	0	-	1	1
Cancer of eye	1	1	0	-	1	1
Cancer of rectum	1	1	0	-	1	1

CIN, cervical intra-epithelial neoplasia; VIN, vulvar intra-epithelial neoplasia.

† , Lymphomas, four non-Hodgkin’s, one central nervous system, one Hodgkin’s, and one immunoblastic.

## Discussion

We found that a quarter of PLWH receiving care at NC were older than 50 years. The majority of these elderly patients had received ART for over 10 years. Most men were married; the majority of women were widowed. This cohort of elderly patients had well-controlled HIV infection with evidence of good immunological status. A high prevalence of overweight or obesity was reported, particularly amongst women. Comorbidities were common, notably hypertension, malignancies (far higher in women than in men) and CKD (higher in men than in women). Nearly 20% of patients had at least two chronic comorbidities, and 5.6% had three.

The goal of ART is sustained suppression of HIV replication. Our results show that elderly patients with HIV achieve high rates of viral suppression. This finding is consistent with the results from other studies.^10^ Older patients receiving ART have demonstrated good medication adherence and this plays a key role in achieving high rates of viral suppression.^14,15^

Although only half of our patients were married at the time of enrolment into care, over 80% of men were married. The higher proportion of marriage amongst men may be because of the fact that men are more likely to remarry after the loss of a spouse or after divorce.^16^ Older patients with HIV face a number of challenges that may affect their quality of life. Loss of partners and friends may lead to social isolation, depression and poor adherence to medicines.^17^ Additional stressors such as unemployment and poverty may further worsen the quality of life. The high number of comorbidities and pill burden may negatively affect medicine adherence. Interactions between ART and other medications are a major consideration amongst the ageing population.

The high prevalence of chronic non-AIDS diseases in this cohort is consistent with the findings from other studies which have showed an increased number of comorbidities in PLWH at all ages, but particularly as this population ages.^6^ Hypertension is now recognised as an important chronic comorbid condition of PLWH and is associated with increased morbidity and mortality.^18^ Screening and optimal management of hypertension must become part of the comprehensive care of elderly PLWH.

The prevalence of CKD was high. Numerous risk factors are involved in renal disease in PLWH, such as age, black race, DM, hypertension, low CD4 counts and certain ART medicines like tenofovir disoproxil fumarate.^19^ All HIV-positive patients especially the elderly must have annual measurements of their renal function. Diabetes mellitus was far less common than hypertension. As has been previously reported, the risk of developing DM in PLWH is not well-established, and studies have not consistently shown a relationship between DM and HIV.^20,21^ However, some studies have shown that the risk of DM increases amongst patients receiving ART.^22^

A large number of elderly patients suffered from different forms of malignancies, which were differently distributed by sex. Human papilloma virus (HPV)-related malignancies were the leading diagnosed cancers. Almost 70% of the women with malignancies had HPV-related CIN3. The HPV-related anogenital malignancies will remain a challenge amongst HIV-infected men and women even in the era of effective ART.^23^ Screening for high-risk HPV infection should become part of the care of HIV-positive women. The estimated cumulative incidence for other cancers such as non-Hodgkin’s B-cell lymphoma, lung cancer, liver cancer and Hodgkin’s lymphoma increased amongst HIV-infected patients.^24^ Routine screening for common cancers should be part of the comprehensive care of HIV-infected elderly patients.

Our study was strengthened by very high levels of data completeness. However, our study was limited as we used data from a single clinic only. Frailty, cognitive impairment and other geriatric syndromes that are common and important in HIV were not included in comorbidities as data on these are not routinely collected. Furthermore, assessing cancers cross sectionally as we did, without consideration of those who have died of cancer prior to the data pull, underestimates the true cancer burden. We assessed all cancers ever diagnosed (historical). We acknowledge that although these data are interesting, they are potentially misleading. Despite these limitations, we believe that our results are important to healthcare providers and policymakers.

## Conclusion

Our data demonstrates that in Africa high rates of virological suppression can be achieved amongst elderly patients on ART. However, the high rates of obesity (especially amongst women) and hypertension in this population need to be addressed. The prevalence of pre-malignant cervical disease amongst elderly women is high; and hence, routine cervical cancer screening is essential for elderly women living with HIV.

The time has come for HIV treatment programmes to draw on a wide range of medical disciplines and evidence-based screening procedures for the multidisciplinary care of the ageing HIV-infected population. Age-appropriate monitoring protocols must be developed to ensure optimum quality of care for elderly HIV-positive individuals.
